# Renal replacement therapy neutralizes elevated MIF levels in septic shock

**DOI:** 10.1186/s40560-016-0163-2

**Published:** 2016-06-16

**Authors:** Julia Pohl, Maria Papathanasiou, Martin Heisler, Pia Stock, Malte Kelm, Ulrike B. Hendgen-Cotta, Tienush Rassaf, Peter Luedike

**Affiliations:** West-German Heart and Vascular Center Essen, Department of Cardiology and Department ofVascular Medicine, University Hospital Essen, Hufelandstr. 55, 45147 Essen, Germany; Medical Faculty, Division of Cardiology, Pulmonology and Vascular Medicine, University Hospital Düsseldorf, Moorenstrasse 5, 40225 Düsseldorf, Germany

**Keywords:** MIF, Septic shock, ICU, Hemodialysis

## Abstract

**Background:**

Macrophage migration inhibitory factor (MIF) is known to amplify the immune response in septic animal models. Few clinical data support this pro-inflammatory role in septic patients. Renal replacement therapy (RRT) as adjuvants in the complex therapy of sepsis has been proposed as a possible approach to eliminate elevated circulating cytokines. Since recent data suggest that MIF can be effectively removed from the circulating blood pool in patients with chronic kidney disease, we here aimed to investigate whether RRT in septic shock can lower plasma levels of this pro-inflammatory cytokine in septic shock patients.

**Methods:**

An observational single-center study on an internist intensive care unit (ICU) was conducted. MIF plasma levels and mortality of *n* = 25 patients with septic shock were assessed with a previously validated method for reliable MIF values. The effect of continuous renal replacement therapy (CRRT) on daily MIF levels and mortality was assessed by comparing patients with and without need for CRRT due to acute kidney injury (AKI).

**Results:**

MIF plasma levels in patients undergoing CRRT due to septic AKI were steadily decreased compared to those from patients without CRRT hinting at a MIF removal by hemodialysis. MIF release during ICU stay as assessed by MIF_AUC_ was lower in patients undergoing CRRT, and Kaplan-Meier analysis revealed a distinctly lower mortality in patients undergoing CRRT. Analysis of daily MIF levels showed that patients who did not survive septic shock exhibited steadily higher MIF plasma levels and higher MIF_AUC_ compared to those surviving sepsis. Low MIF levels were closely associated with improved survival.

**Conclusions:**

This is the first study investigating the effect of efficient MIF removal from the plasma pool of patients with septic shock. Reduction of high circulating MIF by CRRT therapy was accompanied by improved survival. Thus, targeted removal of MIF from the circulating blood pool might be a promising approach to reduce mortality in severe sepsis.

**Electronic supplementary material:**

The online version of this article (doi:10.1186/s40560-016-0163-2) contains supplementary material, which is available to authorized users.

## Background

Severe sepsis and septic shock are major causes of mortality and morbidity worldwide [1]. Septic patients often develop multiple organ dysfunction syndrome (MODS) that is characterized by an acute functional impairment of nearly 50 % of patients presenting with septic shock on an intensive care unit (ICU) develop acute kidney injury (AKI) with the need of (continuous) renal replacement therapy (CRRT) [2]. The activation of multiple pro-inflammatory mediators is the hallmark in the pathophysiology of sepsis. It is widely accepted that hypercytokinemia caused by the inflammatory response to infection and abnormal tissue oxygen metabolism play pivotal roles in the pathophysiology of sepsis [3]. Many of these mediators may directly contribute to organ dysfunction and determine disease severity. The close relationship between high levels of inflammatory cytokines in plasma and mortality in septic patients indicates that the activation of inflammatory mediators plays an important role in the development of organ dysfunction and is directly associated with sepsis-induced AKI. To date, there are attempts to reduce hypercytokinemia using CRRT with consecutive removal of circulating inflammatory mediators in septic patient with AKI. Whether this may be a beneficial intervention during sepsis is under intensive investigation, and first small studies showed improved outcome after cytokine removal in sepsis-induced acute kidney injury [4–8].

*Macrophage migration inhibitory factor* (MIF) is an important mediator of severe sepsis and septic shock [9–11]. MIF is quasi-ubiquitously expressed and stored in numerous cell types, while specifically secreted from the pituitary gland upon endotoxemia [10], from immune cells upon inflammatory stimulation, and from selected endothelial and parenchymal cells upon hypoxic, hyperoxic, and other stress stimuli [12–14]. MIF is a potent upstream regulator of innate immunity through modulation of TLR4 expression, inflammatory cytokine induction, and glucocorticoid overriding effects [15, 16]. MIF was demonstrated to be markedly and persistently up-regulated in patients with gram-negative sepsis and was associated with parameters of disease severity and early death [17].

We recently showed that MIF is a plasma component that can be dialyzed effectively during hemodialysis in chronically ill patients suffering from end-stage renal failure [18]. Whether circulating MIF levels are increased in septic patients with AKI and whether CRRT removes circulating MIF is not known. We here examined 25 patients on an internal medicine ICU admitted due to septic shock concerning the removal of MIF from the circulating plasma pool by continuous veno-venous hemodialysis (CVVHD) and whether this affected mortality in septic patients.

## Methods

### Ethics, consent, and permissions

Ethical approval was obtained from the institutional review board (Ethics commission University Hospital Duesseldorf), and written informed consent by the patients or their spouse was given before participating in the study.

### Study population

Twenty-five patients admitted to an academic, interdisciplinary, internist-neurologic intensive care unit due to septic shock were enrolled. The definition of septic shock was based on criteria established in the Surviving Sepsis Campaign (SSC) guidelines [1]. Patients were divided into two groups according to the presence or lack of acute septic kidney injury with the need for CVVHD.

### Standard treatment of patients

After admission to the ICU and confirmation of septic shock, all patients received standardized intensive care treatment according to the SSC guidelines [1] including fluid substitution, antibiotic treatment, vasopressor treatment, and mechanical ventilation, if necessary. In case of acute respiratory distress syndrome, patients were treated according to a locally standardized protocol adopted the SSC guidelines [19]. Patients were discharged from the ICU after fulfillment of standardized clinical discharge criteria.

### Dialysis specific treatment

The need for CRRT therapy was indicated and approved after medical round by a nephrologist. AKI was defined by the criteria introduced by the Acute Kidney Injury Network in 2012 (increase in creatinine by ≥0.3 mg/dl within 48 h; increase in creatinine to ≥1.5 times baseline, which have occurred within the prior 7 days; or urine volume <0.5 ml/kg/h for 6 h) [20]. CRRT was started based on standardized criteria (anuria, metabolic acidosis, increase of serum creatinine or BUN, increase of serum potassium). Most of the patients underwent dialysis due to anuria and metabolic acidosis. Dialysis was conducted as a continuous veno-venous hemodialysis (CVVHD) using citrate as regional anticoagulant. A detailed description of the citrate CVVHD system has been published earlier [21, 22]. By default, blood flow was 100 ml/min, dialysate flow was 2000 ml/h. Ultrafiltration rate was adjusted flexibly upon clinical requirements and was between 0 and 250 ml/h. Citrate and calcium flow were adjusted flexibly due to plasma and postfilter levels of ionized calcium. High-flux membranes (AV600S, polysulfone membranes, surface area 1.4 m^2^, Fresenius Medical Care, Germany) were used in all patients, and dialysis was performed via standard dialysis catheters inserted into central veins. Cessation of CVVHD treatment was determined after medical round by a nephrologist and was usually conducted due to hemodynamic stabilization and restart of urine production (at least 5 days).

### Blood sample collection

After admission to the ICU and confirmation of the presence of septic shock, the first blood samples were drawn within the first 24 h of ICU admission (first day of ICU treatment) and every following 24 h at the same time of day for maximal 5 days (second, third, fourth, and fifth day of ICU treatment) or until discharge from ICU and death, respectively. Blood samples for MIF measurements were drawn via an arterial catheter into heparinized tubed.

### MIF measurements

Blood samples for determination of MIF plasma levels were centrifuged immediately at 1000×*g* for 15 min at 4 °C. Plasma was obtained and frozen at −20 °C until measurement. MIF plasma levels were determined using an enzyme-linked immunosorbent assay (ELISA, R&D, Minneapolis, USA) as previously described [23–26].

### Data collection

Baseline characteristics were assessed and documented at the first day of enrollment. The simplified acute physiology score (SAPS II) was evaluated on each day [27]. Subsequently, the sequential organ failure assessment (SOFA) score and the Acute Physiology and Chronic Health Evaluation II (APACHE II) score were determined for the daily assessment of organ dysfunction throughout the ICU stay [28, 29]. The clinical course of patients was observed in a follow-up period of 30 days.

### Statistical analysis

All data were statistically analyzed with a commercially available software package (GraphPad Prism 6; GraphPad Software, La Jolla, CA, USA). Data are given as mean and standard error of the mean (SEM) unless indicated otherwise.

Time course of MIF levels was compared by multiple *t* test and Holm-Sidak method for correction of multiple comparisons. The area under the curve of MIF plasma levels (MIF_AUC_) from admission until study drop out (due to discharge, death or end of study) was computed to approach the dynamic and inter-individually different conditions of MIF release expected to occur during ICU stay. MIF_AUC_ was corrected for the number of days of ICU stay to preserve comparability. Survival analysis was done by the Kaplan-Meier method and compared by the log-rank test. The D’Agostino and Pearson omnibus normality test was used to test all data for normal distribution. We used the Student *t* test to compare normally distributed results of single measurements and the Mann-Whitney *U* test to compare non-normally distributed data. Proportions were compared using the chi-square test. In all cases, a level of *P* < 0.05 was considered statistically significant.

### Availability of data and materials

All data presented are available upon request to peter.luedike@uk-essen.de.

## Results

### Characteristics of the study population enrolled at the intensive care unit

Twenty-five patients with septic shock were included in this prospective study. Sepsis foci were pulmonary infections (72 %), urogenital infections (16 %) or other sites of infection (12 %). In 10 patients out of 25, a microbiological pathogen could be identified. Comparing septic patients with (*n* = 11) and without the need for CRRT (*n* = 14), neither site of infection nor ICU scores (APACHE II, SOFA, SAPS II) depicting disease severity or laboratory values were significantly different (Table 1). However, creatinine levels at admission to the ICU were higher in patients needing CRRT (2.5 ± 0.2 vs. 1.4 ± 0.2 mg/dl, *n* = 11–14, *P* < 0.01, Table 1) indicating a higher incidence of AKI in this group. Basic demographic and clinical and biochemical characteristics are summarized in Table 1.

**Table 1:** Patients’ characteristics

	Total (*n* = 25)	No CRRT (*n* = 14)	CRRT (*n* = 11)	*P* value
Age (years)	73 ± 3	74 ± 4	73 ± 4	0.7946
Male (%)	80	85	72	0.5127
APACHE II	26 ± 2	25 ± 2	28 ± 3	0.4645
SAPS II	41 ± 2	39 ± 3	43 ± 3	0.4815
SOFA	9 ± 1	9 ± 1	9 ± 1	0.7636
Mechanical ventilation (%)	76	79	73	0.7872
Need for vasopressors (%)	100	100	100	1
Sites of infection (%)				
Lung	72	86	55	0.0849
Urogenital	16	14	18	0.7350
Other	12	7	27	0.7341
Microbiological data (*n*)				
Gram-negative/positive	3/7	3/4	0/3	0.4750
Blood urea nitrogen (mg/dl)	77 ± 8	70 ± 9	86 ± 14	0.3497
Creatinine (mg/dl)	1.8 ± 0.2	1.4 ± 0.2	2.5 ± 0.2	<0.01
WBC (/μl)	11.7 ± 1.4	11.9 ± 2	11.4 ± 2	0.8834
CRP (mg/dl)	16.8 ± 2.6	21 ± 4	12 ± 2	0.1447
PCT (ng/ml)	8.7 ± 3.4	7.8 ± 2.5	10.4 ± 8.8	0.4982
Co-morbidities (%)				
Coronary artery disease	28	33	18	0.3895
NYHA IV	4	0	9	0.2496
COPD	27	24	9	0.0620
Pulmonary hypertension	8	7	9	0.8586
Immunosuppression	23	21	27	0.7341
Hepatic disease	8	7	9	0.8586
Reasons for CVVHD (%)				
Anuria/oliguria			81	
Hyperkalemia			18	
Acidosis			18	

### MIF removal from circulating plasma pool of septic patients by CVVHD is associated with an improved outcome

**Fig. 1:**
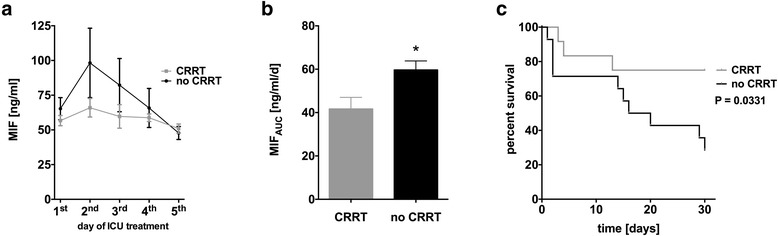
MIF removal from circulating plasma pool of septic patients by CVVHD is associated with an improved outcome. **a** Septic patients undergoing CRRT due to septic acute kidney injury showed lower MIF levels compared to septic patients without the need for CRRT during ICU stay. **b** Total MIF release during ICU stay as assessed by MIF_AUC_ was lower in patients undergoing CRRT compared to those without the need for CRRT. **c** Kaplan-Meier survival curves show increased 30-day mortality for patients without CRRT compared to those undergoing CRRT

During ICU treatment, patients undergoing CRRT due to septic acute kidney injury showed lower MIF levels compared to septic patients without the need for CRRT. The maximum of plasma MIF was reached 24 h after ICU admission in patients not undergoing CRRT, while there were no differences in MIF levels during CRRT (Fig. 1a). MIF levels equalized at day 5 (Fig. 1a). MIF_AUC_ as a parameter for total amount of circulating MIF during ICU stay was significantly lower in patients undergoing CRRT compared to those without the need for CRRT (41.7 ± 5.4 vs. 59.6 ± 4.2 ng/ml, *n* = 11–14, *P* = 0.05, Fig. 1b) demonstrating effective MIF removal by CRRT. Importantly, MIF levels at admission did not vary between patients with need for CRRT and those without (data not shown). Kaplan-Meier surviving curves showed improved survival for patients undergoing CRRT and with consecutive lower MIF_AUC_ (*P* = 0.0331, log-rank = 4.539) compared to septic patients without AKI and without CRRT. Efficacy of CRRT treatment was proofed by decrease of creatinine levels (Additional file 1: Figure S1).

### Sustained lowered MIF levels are associated with decreased mortality in septic patients

**Fig. 2:**
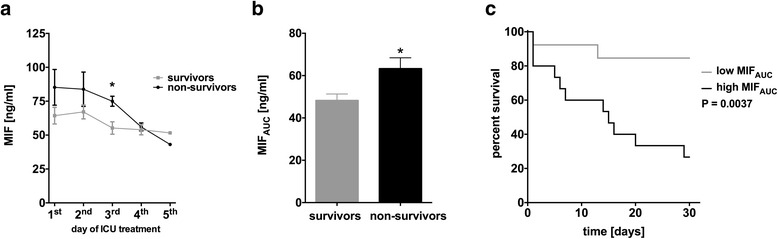
Sustained lowered MIF levels are associated with decreased mortality in septic patients. **a** Survivors of septic shock showed lower MIF plasma levels during ICU stay compared to non-survivors. **b** Total MIF release was significantly lower in patients who survived septic shock. **c** Kaplan-Meier survival curves are displayed, showing that patients with high MIF (MIF_AUC_ >51.9 ng/ml) had an increased short-time mortality at the ICU compared to those with low MIF (MIF_AUC_ <51.9 ng/ml) during ICU stay

In patients who survived septic shock, MIF plasma levels during ICU stay were lower compared to patients who did not survive septic shock (Fig. 2a). MIF release during ICU stay was significantly lower in patients who survived septic shock as depicted by MIF_AUC_ (48.3 ± 3 vs. 63.3 ± 5.1 ng/ml, *n* = 11–14, *P* < 0.05, Fig. 2b). High MIF_AUC_ (beyond median, >51.9 ng/ml) was associated with increased 30-day mortality compared to MIF_AUC_ (below median, <51.9 ng/ml) as shown by Kaplan-Meier surviving curves (*P* = 0.0037, log-rank = 8.43, Fig. 2c).

## Discussion

Severe sepsis and septic shock are major health care problems, affecting millions of people around the world and killing at least one in four [1]. Improving diagnosis, optimizing sepsis therapy and thereby limiting high mortality is the goal of the surviving sepsis campaign. Renal replacement therapy belongs to supportive sepsis therapy approaches and should be conducted as CRRT to facilitate management of fluid balance and hemodynamic stabilization [1]. In first small studies, additional effects of hemodialysis during septic shock have been investigated. Besides general immunomodulatory effects, it has been described that CRRT can be used as a cytokine modulator since first studies showed that removal of various pro-inflammatory cytokines such as TNFα, IL-6 and IL-8 from the bloodstream resulted in early recovery from septic shock [30–32].

MIF is a key mediator of severe sepsis and septic shock [9–11], and it was demonstrated that MIF levels are persistently elevated in patients with sepsis, and high MIF levels were associated with parameters of disease severity and early death [17]. In experimental sepsis models, MIF inhibition by MIF antibodies and MIF receptor antagonists was shown to protect from septic shock and to improve survival [9, 33, 34]. We recently showed that hemodialysis is an effective tool to neutralize circulating MIF by removal from the blood pool in patients with end-stage renal failure [18]. In the present study, we show first evidence of the effect of lowering MIF levels in patients undergoing CRRT in septic shock.

Baseline characteristics of these patients did not differ except for retention parameters (creatinine) indicating a higher incidence of AKI in CRRT group as expected. The measured MIF values in patients with CRRT were steadily lower compared to patients without CRRT until reaching a steady state at day 5 hinting at a possible removal by CRRT. Of course, our study remains as a proof of concept, since the exact mechanism of MIF removal by CRRT has not been addressed in this study. Our results are based on isolated measurements of plasma MIF levels, which reflect a sum of circulating, newly produced, secreted, and removed as well as degraded MIF. With our data, it is not possible to distinguish between production, removal, and degradation rates of MIF. Nonetheless, it is unlikely that the differences in MIF levels are due to different production rates. It is more likely to assume that MIF levels are lowered by CRRT since conduction of CRRT is the only obvious MIF-influencing difference between our groups. Furthermore, our group proofed effective removal of MIF by dialysis before [18]. In addition, the comparison of decrease of MIF levels with decrease of creatinine levels during CRRT showed a close correlation (Additional file 2: Figure S2) hinting at a similar mechanism.

Overall total amount of circulating MIF during ICU stay as measured by MIF_AUC_ was significantly lower in CRRT patients indicating effective removal by hemodialysis. There is lack of knowledge concerning MIF releasing cell type, MIF plasma half life and its relation to MIF removal in patients with septic shock. Furthermore, blood purification is a complex topic and intensive studies are needed to confirm MIF removal and to calculate MIF clearance by CRRT. Nevertheless, our study shows first evidence that CRRT in septic shock modulates not only TNFα, IL-6, and IL-8 levels but also MIF levels to reduce hypercytokinemia with a close correlation to improved survival.

Proof of effective MIF removal gives us two new viewpoints on the role of MIF in sepsis: MIF removal by CRRT is an option to neutralize MIF in septic shock and since MIF receptor antagonists and MIF antibodies are not approved for human use yet, CRRT is the only option for MIF neutralization in humans. None of existing studies on the role of MIF in sepsis report the overall incidence of CRRT in the investigated patients, and none of these studies scrutinized whether CRRT might influence and confound measured MIF levels.

Moreover, patients receiving CRRT and exhibiting decreased MIF levels showed improved survival. Since 47.5 % (95 % CI, 45.2–49.5 %) of patients presenting with septic shock on ICU develop acute kidney injury with the need of CRRT [2], this issue is of great importance. Mortality rates in patients with septic shock are known to be extraordinarily high. In the present study, mortality rates showed two peaks with one at a very early time point and one at around day 10 after admission (Fig. 1c). Whether this late rise in mortality can be attributed to the increased MIF levels in this group cannot be answered yet. Since the development of systemic inflammatory response syndrome and subsequent development of multiple organ dysfunction syndrome appear to be related to MIF levels and the balance of Th1 and Th2 function, this might be an explanation for the observed kinetic [35].

Of note, our study has some limitations. It is limited by its observational approach without providing a prior power analysis that allows for a distinct statement on the role of MIF removal by CRRT on mortality. Besides, a major limitation of this study is the small number of patients. Larger studies are needed to confirm MIF neutralization and its effects on improved survival. As mentioned before, this study did not elucidate the mechanism of potential MIF removal by CRRT. Despite these limitations considering the study design, we can draw three clear-cut messages from this study.

First, MIF plasma levels can be effectively decreased by CRRT in septic patients on an ICU. Second, further studies have to consider CRRT as a critical confounder concerning the measurement and interpretation of MIF levels since removal of MIF must be taken into consideration. Third, reduced MIF levels are associated with improved survival of septic shock, and therefore, the removal of MIF could be a new therapeutic approach for adjunctive sepsis therapy.

## Conclusions

In summary, we here provide first evidence to effectively influence MIF plasma levels by CRRT in septic patients with AKI. CRRT with consecutive lowering of MIF levels was closely associated with survival in those patients and these preliminary results should be taken into consideration further studies to develop novel concepts of adjunctive sepsis therapy.

## Additional files

Additional file 1: Creatinine values. (TIFF 155 kb) Additional file 2: Correlation of MIF and creatinine reduction during renal replacement therapy. (TIFF 166 kb)

## Abbreviations

AKI: acute kidney injury
CRRT: continuous renal replacement therapy
CVVHD: continuous veno-venous hemodialysis
ICU: intensive care unit
MIF: macrophage migration inhibitory factor
