# Supplementary figures and images for: Renal replacement therapy neutralizes elevated MIF levels in septic shock
Source: J Intensive Care. 2016 Jun 16;4:39. doi: 10.1186/s40560-016-0163-2 (PMC4910205; doi:10.1186/s40560-016-0163-2)

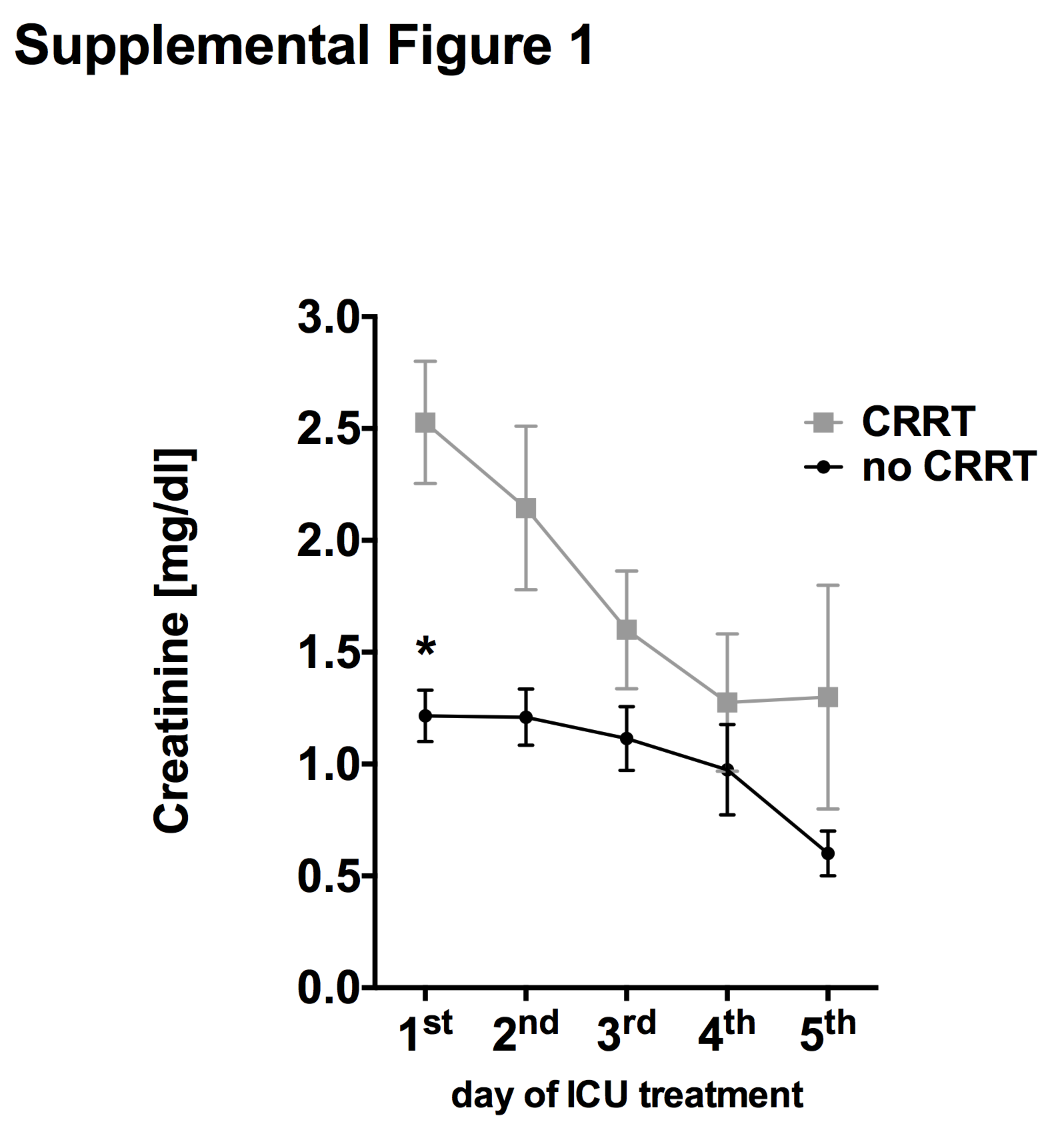

Supplement: Additional file 1: — Creatinine values. (TIFF 155 kb) [file 40560_2016_163_MOESM1_ESM.tiff]

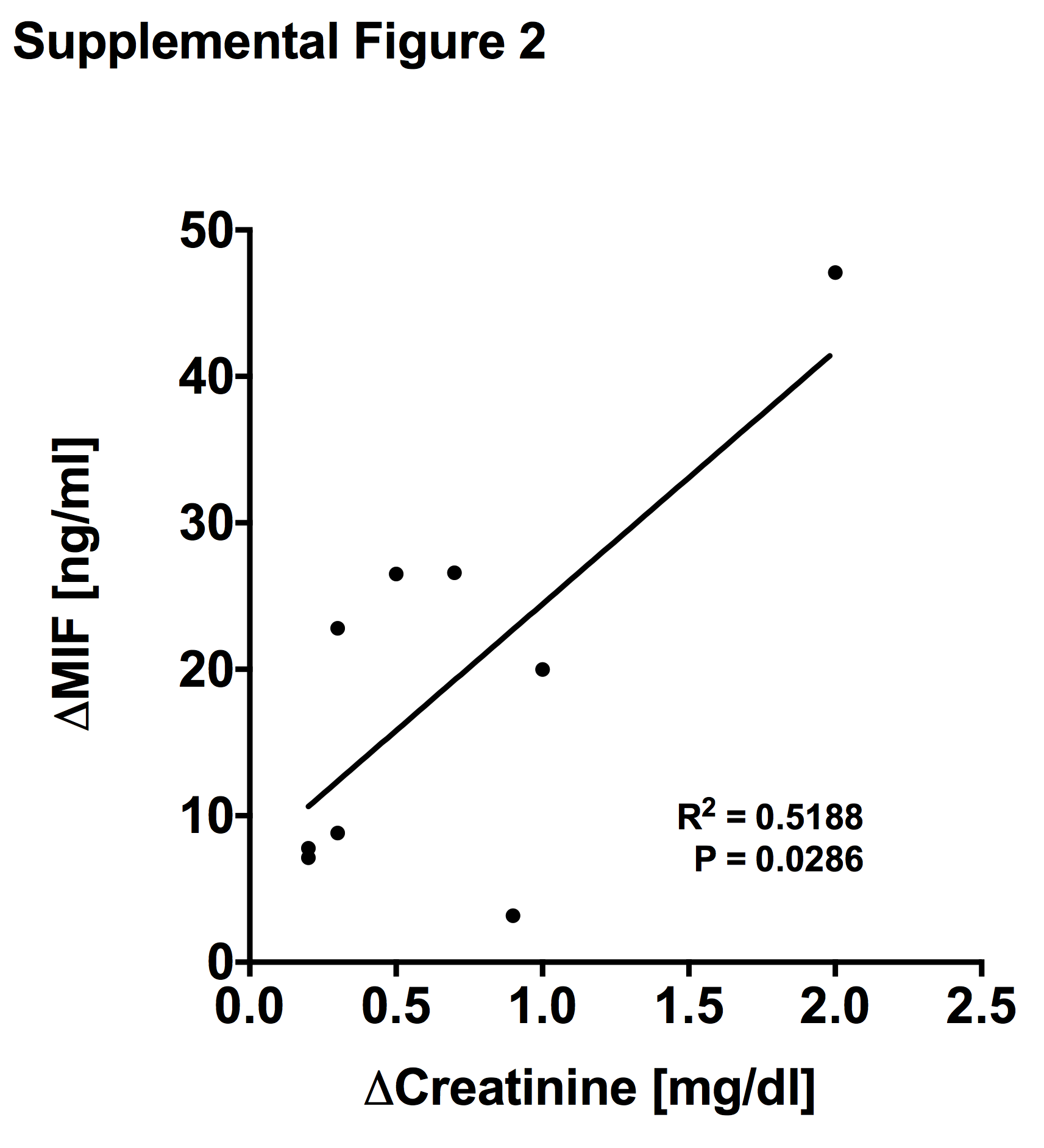

Supplement: Additional file 2: — Correlation of MIF and creatinine reduction during renal replacement therapy. (TIFF 166 kb) [file 40560_2016_163_MOESM2_ESM.tiff]
